# Effect of Gut Microbiota Biotransformation on Dietary Tannins and Human Health Implications

**DOI:** 10.3390/microorganisms9050965

**Published:** 2021-04-29

**Authors:** Ibrahim E. Sallam, Amr Abdelwareth, Heba Attia, Ramy K. Aziz, Masun Nabhan Homsi, Martin von Bergen, Mohamed A. Farag

**Affiliations:** 1 Pharmacognosy Department, Faculty of Pharmacy, October University for Modern Sciences and Arts (MSA), 6th of October City 12566, Egypt; iezz@msa.eun.eg; 2 Chemistry Department, School of Sciences & Engineering, The American University in Cairo, New Cairo 11835, Egypt; amr.abdelwareth@aucegypt.edu; 3 Department of Microbiology and Immunology, Faculty of Pharmacy, Cairo University, Cairo 11562, Egypt; heba.mohamed@pharma.cu.edu.eg (H.A.); ramy.aziz@gmail.com (R.K.A.); 4 Microbiology and Immunology Research Program, Children’s Cancer Hospital Egypt 57357, Cairo 11617, Egypt; 5 Helmholtz-Centre for Environmental Research-UFZ GmbH, Department of Molecular Systems Biology, 04318 Leipzig, Germany; masun.homsi@ufz.de; 6 Institute of Biochemistry, Faculty of Life Sciences, University of Leipzig, Talstraße 33, 04103 Leipzig, Germany; 7 Pharmacognosy Department, Faculty of Pharmacy, Cairo University, Cairo 11562, Egypt

**Keywords:** tannins, gut microbiota, mutual interaction, polyphenols, biotransformation, molecular motifs

## Abstract

Tannins represent a heterogeneous group of high-molecular-weight polyphenols that are ubiquitous among plant families, especially in cereals, as well as in many fruits and vegetables. Hydrolysable and condensed tannins, in addition to phlorotannins from marine algae, are the main classes of these bioactive compounds. Despite their low bioavailability, tannins have many beneficial pharmacological effects, such as anti-inflammatory, antioxidant, antidiabetic, anticancer, and cardioprotective effects. Microbiota-mediated hydrolysis of tannins produces highly bioaccessible metabolites, which have been extensively studied and account for most of the health effects attributed to tannins. This review article summarises the effect of the human microbiota on the metabolism of different tannin groups and the expected health benefits that may be induced by such mutual interactions. Microbial metabolism of tannins yields highly bioaccessible microbial metabolites that account for most of the systemic effects of tannins. This article also uses explainable artificial intelligence to define the molecular signatures of gut-biotransformed tannin metabolites that are correlated with chemical and biological activity. An understanding of microbiota–tannin interactions, tannin metabolism-related phenotypes (metabotypes) and chemical tannin-metabolites motifs is of great importance for harnessing the biological effects of tannins for drug discovery and other health benefits.

## 1. Introduction

Tannins are water-soluble polyphenols with molecular weights ranging between 120 and 3000 Da. These plant secondary metabolites are so named because of their ability to precipitate proteins and thus stabilise animal skin protein against putrefaction during leather manufacture [1]. Additionally, these metabolites have the ability to precipitate alkaloids, gelatine and other proteins; thus, they are often termed antinutrients [2]. In recent decades, the isolation and identification of bioactive stilbenoids from spruce tree bark, as well as various resveratrol oligomers and phlorotannins from brown algae, have expanded the range of known tannins by incorporation of the category of tannin polyphenols [3].

Owing to their polyphenolic structures, tannins have been the subject of various epidemiological studies and were reported to exhibit a myriad of pharmacological effects, such as anti-inflammatory, antioxidant, anticancer, antidiabetic and antimicrobial effects [4,5].

A major limitation of studies investigating the pharmacological activity of polyphenols is their pharmacokinetics. The in vivo bioavailability of these compounds is poor owing to multiple factors, including the high molecular weights of condensed polymers and interactions with other dietary constituents, such as minerals and fibres [6,7]. Tannins are major phenolics in several dietary sources that are not subjected to human metabolism owing to their polymeric nature. Instead, they are mostly metabolised under the action of the gut microbiota, and this metabolism has not been comprehensively reviewed. In the gut, tannins are metabolised by microbial enzymes, and the combined in vivo activity of enteric and hepatic enzymes leads to the formation of conjugated derivatives, which have different pharmacological profiles and are rapidly excreted through urine or via bile secretions back into the gastrointestinal tract (GIT) [6].

Consequently, these pharmacokinetic and metabolic effects question the relevance of in vitro studies investigating the effects of polyphenols on cell lines. The following factors may raise concerns with in vitro-only studies: First, a given tested compound may not be applied in an isolated form in vivo; thus, the pharmacological outcomes may be the concerted effects of mixtures of components in plant extracts rather than single components. Second, the initial concentration and form of the test component may be different from those reaching the target cell in vivo. Third, the interaction time with the target cell to produce pharmacological effects may vary according to in vitro experimental time. Fourth, in vitro studies disregard other interfering factors, such as other food components and the effect of in vivo metabolism, such as the activity of hepatic conjugating enzymes [6]. In sum, although in vitro studies are quite useful for preliminary investigation, they tend to be reductionist and oversimplified, whereas the in vivo situation is often complex and requires systems-level analysis and contextual interpretation.

The relationships between the biological effects of gut-biotransformed metabolites of tannins and molecular profiling data is largely unexplored. It is therefore desirable to find ways to bridge the gap between their chemical structures and their potential mechanism of action, thereby facilitating the discovery of localised molecular features that produce the heterogeneous information of a pharmacological effect. Explainable artificial intelligence (XAI) has been widely used in the drug discovery domain, from molecular design to macromolecular target identification [8]. In this article, XAI is used to identify chemical features of tannin metabolites to guide the prediction of four trained machine learning (ML) algorithms for anti-inflammatory, antioxidant, anticancer and anti-atherosclerotic effects.

Accordingly, evaluation of the pharmacological potential of polyphenols, including tannins—either originally ingested or metabolically generated—should be based on a thorough understanding of their pharmacokinetic and metabolic profiles. The aim of this review article is to delineate the metabolic diversity of tannins, with special emphasis on the mutual interactions of tannins with the colonic microbiota, to review the chemopreventive actions of tannins, to review the microbiota-mediated biotransformed metabolites of tannins and, finally, to highlight the correlation between the chemical substructures of tannin metabolite and their consequential health effects.

The strategy adopted in this article is to delineate the chemical structure and varieties of either hydrolysable or condensed tannins, mention their natural sources and abundance, describe their absorption and metabolism by gut microbiota-related metabolites, discuss the reverse effect of tannins or their metabolites on the microbial community itself and, finally, identify past research findings considering the pharmacological effects of tannin metabolites on the host, with emphasis on microbiota-mediated metabolites.

## 2. Chemistry and Diversity of Tannins

The chemical structures and concentrations of tannins greatly vary among plant species. Several factors, such as growth stage and conditions, including temperature, light intensity, nutrients and exposure to herbivores, affect tannin abundance [9]. Based on their phenolic cores, tannins are chemically classified as hydrolysable tannins (HTs), condensed tannins (CTs) and phlorotannins. Tannins are widely distributed in plants and are especially abundant in forages, shrubs, cereals and other herbs. Additionally, tannins are found in tea and in many fruits, such as bananas, berries, apples and grapes, especially at unripe stages. While HTs and CTs can be found in terrestrial plants, phlorotannins only occur in marine algae [3]. Table 1 lists certain plants reported to be rich in tannins and their classes.

### 2.1. Hydrolysable Tannins

HTs are named so because they are hydrolysed by acids, bases or esterase enzymes into sugars and gallic acid, which in turn can yield the hepatotoxic and irritant compound pyrogallol. Chemically, HTs are made up of a polyol core, consisting mainly of glucose esterified with phenolic, gallic and ellagic acids, with molecular weights ranging from 500 to 3000 Da. These tannins are further classified as gallotannins (GTs) and ellagitannins, which upon hydrolysis yield gallic and ellagic acids, respectively, in addition to sugar moieties [17]. Despite being the most abundant polyol molecule identified in tannins, glucose is not the only molecule that contributes to the galloylation process. Other sugar molecules have been identified as contributors to this process, such as hamamelose and saccharose, as well as other organic acids and alcohols, e.g., quinic acid and quercitol. Nevertheless, these derivatives are rare in nature and have only been reported in a few plants, such as maple, chestnut and witch hazel [18].

**Figure 1:**
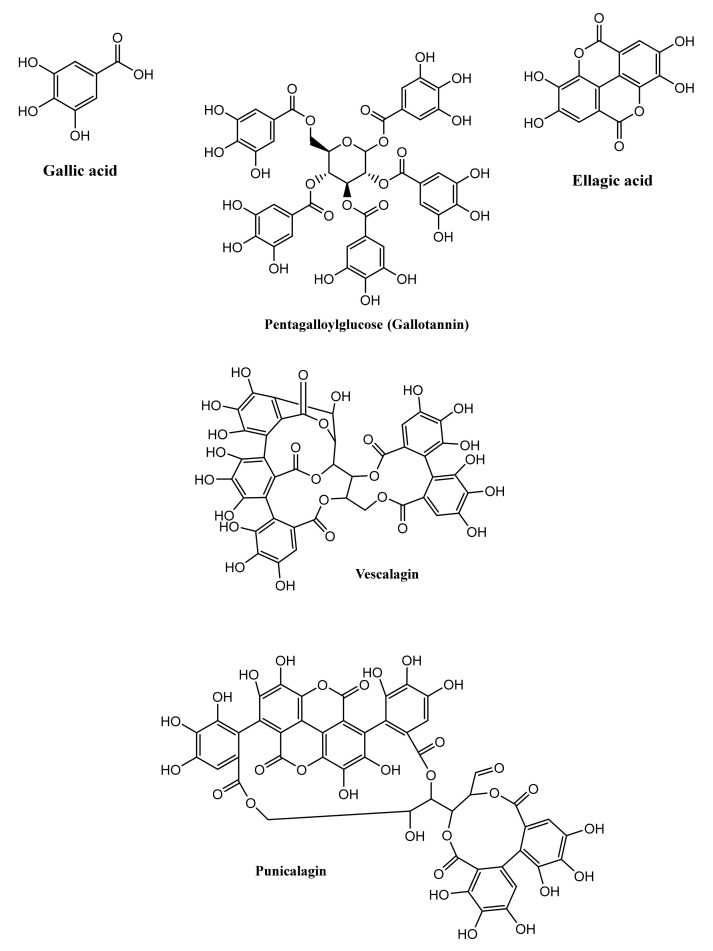
Representative examples of hydrolysable tannins in nature.

GTs are considered the simplest form of HTs. These tannins contain a polyol residue, mainly D-glucose in a β configuration, which is attached to a gallic acid moiety to initially form β-glucogallin (1*-O-*galloyl-β-D-glucopyranose). Furthermore, galloylation yields di-, tri-, tetra-, penta-, hexa-, hepta- and octagalloylglucoses, of which 1,2,3,4,6-penta-*O*-galloyl-β-D-glucopyranose is considered the prototypical GT molecule (Figure 1) [17]. These esterification reactions usually involve the aromatic hydroxy groups, with subsequent formation of meta- or para-depside bonds; however, other GT derivatives, with ten or more gallic acid units esterified to a single glucose moiety, are found in nature, such as those in *Rhus semialata* (sumac) or *Quercus infectoria* (oak galls) [14]. Although the β configuration of the glucose moiety is the most dominant form in GTs, rare examples of the α configuration are also found in nature. This low abundance could be attributed to the low abundance of α glucose compared to that of the β form [2].

Ellagitannins, unlike the rarely found GTs, are highly abundant in many plant species and families. More than 500 ellagitannin molecules have been reported. Although these tannins are classified as HTs, not all ellagitannins are hydrolysable; however, they are still classified as HTs for historical reasons [19]. Ellagitannins can be formed from GTs via intermolecular carbon–carbon coupling between two galloyl moieties to yield at least hexahydroxydiphenic acid, which spontaneously lactonises to gallic acid in aqueous solution (Figure 1). Ellagitannins exist in different monomeric (e.g., punicalagin), dimeric (e.g., sanguiin), oligomeric (e.g., nupharin) and *C*-glycosidic (e.g., vescalagin and castalagin) forms [13].

### 2.2. Condensed Tannins

**Figure 2:**
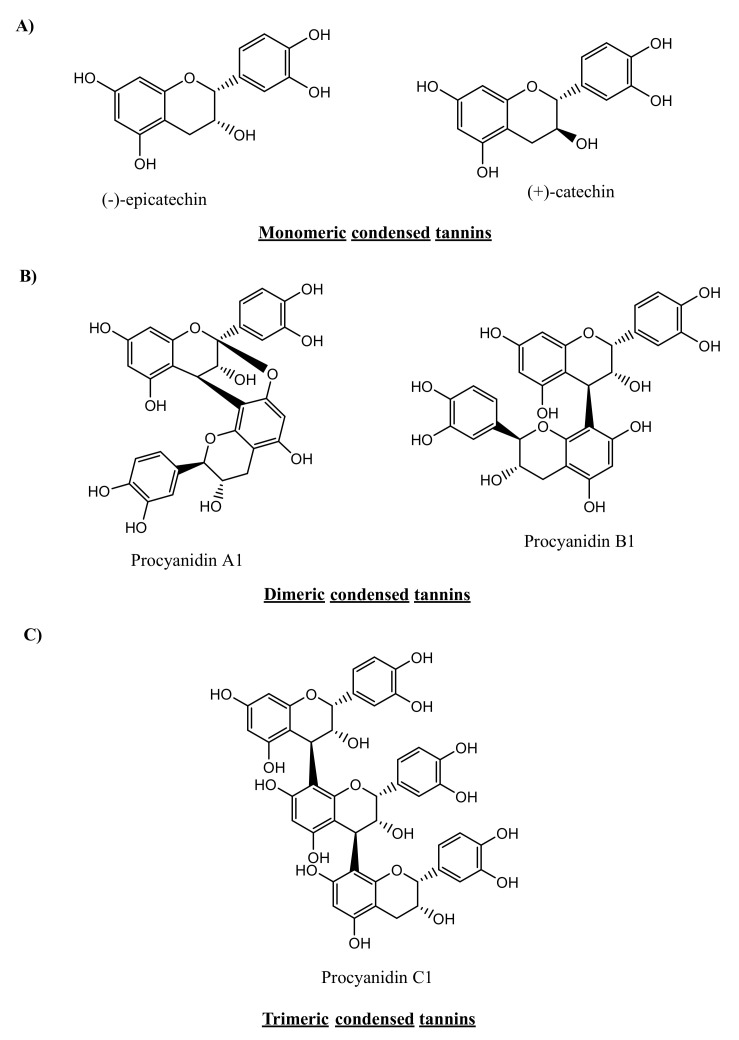
Representative examples of condensed tannin structures in nature for monomeric (**A**), dimeric (**B**) and trimeric (**C**) tannin types.

CTs are plant secondary metabolites that constitute a subgroup of flavonoids. CTs have many synonyms, such as proanthocyanidins or flavan-3-ol polymers. These tannins constitute an important part of plant polyphenols and were first isolated by the French scientist, Jacques Masquelier [20]. CTs, in their monomeric form, are derived from the flavan-3-ol molecule, which consists of two aromatic rings linked via a three-carbon chain (C6-C3-C6, Figure 2A). Oligomeric and polymeric forms of CTs are composed of monomeric flavan-3-ol units and acquire highly complex chemical structures and, consequently, high molecular weights, ranging from 1000 to 20,000 Da [16]. Unlike HTs, CTs can be depolymerised only by strong acidic and oxidative hydrolysis and are not affected by anaerobic enzymatic degradation [21].

In nature, CTs are present as monomers, oligomers (condensed 2–3 or 4 monomer units) or polymerised molecules with high molecular weights. Polymerisation occurs through the C4-C8 or C4-C6 linkage of two monomeric units of flavan-3-ol. The naming convention of the formed polymers varies based on the structure of the monomeric subunit and the linkage position (see Figure 2B for dimers and Figure 2C for trimers). The flavan-3-ol at the end of the proanthocyanidin polymer is called the terminal unit, whereas other monomers in the molecule are called extension units [22].

Proanthocyanidin dimers are classified into dimeric B-type proanthocyanidin and the less abundant dimeric A-type proanthocyanidin. A-type proanthocyanidin has an additional glyosidic linkage at the C2-C7 position. Notably, procyanidin B1 and B2 can be converted to A1 and A2, respectively, via radical oxidation using 1,1-diphenyl-2-picrylhydrazyl (DPPH) radicals under neutral conditions [23]. Whether such conversion can also be enzymatically achieved remains to be reported.

Trimeric forms of procyanidin exist in the form of C1 and C2 trimers. Procyanidin C1 is an epicatechin trimer in which monomeric units are linked via 4β-8 linkage. Procyanidin C2 is a catechin trimer bonded via 4α-8 linkage.

This structural diversity of CTs allows them to exert many reported biological activities. For example, CTs from the barks of *Clausena lansium* possess anti-α-glucosidase, antityrosinase, antiproliferative and apoptotic activities [24]. Another reported activity is the antioxidant effect of fractions containing a high content of CTs from *Sorghum bicolor* [25]. Apple CTs exert hypocholesterolemic properties imparted by their direct interactions with cholesterol via ionic and hydrophobic interactions, as well as intermolecular hydrogen bonding [25]. Moreover, CTs from different plant sources were reported to exhibit antimicrobial activities, such as the inhibitory effect of rice straw CTs on *Staphylococcus aureus*, through the reduction of intercellular ATP and inhibition of bacterial biofilm formation [26]. Likewise, CT derivatives assembled with polysaccharides exhibited an antiadhesive and bactericidal effect on *Pseudomonas aeruginosa* and *Staphylococcus aureus*, which suggested their use to avoid infections imparted by bacterial adhesion to biomedical appliances [27]. Finally, CTs from *Leucaena leucocephala* showed apoptotic activity against human breast adenocarcinoma (MCF-7), human colon carcinoma (HT29), human cervical carcinoma (HeLa) and human liver carcinoma (HepG2) cell lines in vitro through cell shrinkage and nuclear condensation [28].

## 3. Metabolism of Tannins

### 3.1. Non-Microbiota-Mediated Metabolism of Tannins

#### 3.1.1. Hydrolysable Tannins

Tannin bioavailability, and thus their consequent pharmacological actions, is greatly influenced by the tannin absorption rate, in addition to possible metabolism by the gut microbiota or liver enzymes [29]. A few studies have reported the enzymatic digestion of HTs to the corresponding monomers, gallic or ellagic, in the stomach or small intestine. Konishi, Hitomi and Yoshioka [30] reported that upon oral administration, intestinal absorption of gallic acid was relatively slow, with a t_max_ of 60 min in rats [30], while Shahrzad and coworkers reported comparable results in humans, with a t_max_ of 1.27 h [31]. Moreover, other studies reported two different pathways for gallic acid absorption in the stomach and small intestine (detailed in [31]).

In general, the intact gallic acid moiety adopts a rapid permeation system, while its derivatives adopt a slow system [32]. For ellagitannins, Seeram et al. [33] reported the detection of free ellagic acid in the plasma of individuals post 0.5 and 3 h of oral administration of pomegranate juice containing 24 mg of ellagic acid and 318 mg of ellagitannins, while no intact ellagitannins were detected [33]. Similar results were obtained in rats by Lei et al. [34]; however, in both studies, the plasma levels of free ellagic acid were relatively low, owing to the moderate solubility of this compound in water. Other factors that contribute to such low plasma levels of free ellagic acid are its irreversible binding to DNA and proteins, and consequently its limited transcellular absorption, as well as the formation of poorly soluble complexes with metals in the intestine [35].

After absorption, the free ellagic acid undergoes further conjugation reactions with methyl, glucuronyl or sulfate groups, and these conjugates have indeed been detected in human urine and plasma [12].

#### 3.1.2. Condensed Tannins

Only 5–10% of the ingested polyphenols are estimated to be absorbed intact from the small intestine. Flavan-3-ol monomers are absorbed intact from the small intestine. Several human and animal studies showed that (+)-catechin and (−)-epicatechin were rapidly absorbed from the upper portion of the small intestine. The maximum (+)-catechin levels in human plasma were reached, 1.4 h after the intake of dealcoholised red wine [36]. However, recent studies indicated that, in terms of bioavailability, the rate of absorption of flavan-3-ol monomers varies according to chemical structure and stereochemistry in the order (−)-epicatechin > (+)-epicatechin or (+)-catechin > (−)-catechin, which is suggestive of a stereospecific enzymatic process or receptor-mediated transport [37].

These compounds are further absorbed into the blood stream in the aglycone form and further metabolised, first by cells of the small intestine and then in the liver. Systemic metabolism of flavan-3-ol compounds may occur via glucuronide formation, sulfonation or methylation. Catalysed by uridine diphosphate glucuronosyltransferases (UGTs), glucuronide formation occurs in the luminal part of the endoplasmic reticulum, while sulfation and methylation are conducted in the cytosol by sulfotransferases (SULTs) and catechol-*O*-methyltransferases (COMTs), respectively [38].

While the small intestine is the main site of glucuronidation, the liver is considered the main site for sulfonation or methylation of flavan-3-ol monomers or their microbial metabolites [23]. Notably, the stereochemistry of flavan-3-ol monomers not only affects their bioavailability rate, but also has an impact on the rate and type of in vivo conjugation. For example, Ottaviani and coworkers [39] reported that the bioavailability of epicatechin-3′-*O* -glucuronide and epicatechin-3′-sulfate was higher upon administration of (−)-epicatechin than upon administration of (+)-epicatechin, which was not the case with epicatechin-5-sulfate [39].

Epicatechin-3′-*O*-sulfonate and epicatechin-3′-*O*-glucuronide are the major metabolites detected in urine, plasma and bile after administration of (−)-epicatechin [40]. However, other metabolites were also identified in plasma and urine after in vivo administration of a cocoa-based drink [39] and dark chocolate [41]. These metabolites include 4′-*O*-methyl-epicatechin-7-*O* -glucuronide, (−)-epicatechin-5-sulfate, (−)-epicatechin-7-sulfate, 3′-*O* -methyl-epicatechin-5-sulfate, 4′-*O* -methyl-epicatechin-5-sulfate, 3′-*O* -methyl-epicatechin-7-sulfate, 4′-*O* -methyl-epicatechin-5-sulfate, (−)-epicatechin-4′-*O* -glucuronide and (−)-epicatechin-4′-sulfate. The absence of (−)-epicatechin as the sole product of methylation indicates that either glucuronidation or sulfonation by enterocytes and hepatocytes is a prerequisite for the occurrence of methylation in human subjects. On the other hand, the glucurisation position of tea catechins, epigallocatechin (EGC) and epigallocatechin gallate (EGCG) was shown to affect their degree of methylation [42]. While glucurinisation of the A ring had no inhibitory effect on methylation, glucurinusation at either B or D rings inhibited the same ring methylation to a great extent [42].

Borges et al. identified these Phase II metabolites as structurally related (−)-epicatechin metabolites and identified these metabolites as being responsible for the first peak of the biphasic plasma bioavailability profile of flavan-3-ol monomers, which occurs approximately one hour after oral ingestion. Upon administration of bioactive 2-^14^C labelled epicatechin, 82% of the radioactivity was recovered from plasma and urine, 12.3% was expelled with faeces, and 5.2% was present in tissues [43].

Proanthocyanidin dimers are also absorbed directly through the upper GIT. The absorption rate of intact proanthocyanidin dimers is 5–10% that of epicatechin monomers [22]. It is estimated that 2% of procyanidin dimers are absorbed via the GIT 3 h post ingestion. The reduced rate of dimer absorption is attributed to complex formation with salivary proteins, which hinders the absorption of the dimers form [44].

Proanthocyanidin dimers, trimers and tetramers show a decreased rate of absorption concurrent with their increasing molecular size and the number of hydrophilic hydroxyl groups. The absorption of these complexes occurs via paracellular diffusion rather than active transport through Caco-2 cells [16]. Polymer metabolism by Phase II enzymes appears to be weaker than monomer metabolism [22]. However, minute fractions of procyanidin dimers, such as B1, B2 and B5, have been detected in plasma or urine after cocoa and grape seed intake.

Apart from monomers, no Phase II enzyme metabolites have been detected in bodily fluids, except for some methyl derivatives [23]. Proanthocyanidin polymers of more than four subunits are not absorbed intact. All non-absorbed proanthocyanidins (up to 95% of tannin intake) reach the colon intact, where these compounds are metabolised by the colonic microbiota to yield various metabolites. This group includes flavan-3-ol oligomers with three or more monomeric units. These oligomers, along with bile-excreted Phase II metabolites, constitute the substrates for further metabolism by colonic microbes [45].

### 3.2. Microbiota-Mediated Metabolism of Tannins

#### 3.2.1. Composition of the Gut Microbiota

The human gut is the natural habitat for a complex microbial community, comprising bacteria, archaea and eukaryotes that have evolved to live on the intestinal mucosal surfaces and lumen, in addition to viruses/phages that infect them. The gut microbiota includes trillions of microorganisms, which outnumber the human nucleated cells by an order of magnitude [46]. Approximately 10^14^ bacterial cells colonise the GIT but are unequally distributed, with the highest numbers in the large intestine, representing the largest community of microorganisms that has been reported to date in a single environment. [47].

Soon after birth, the human large intestine starts to host a broad spectrum of highly metabolically active species. A vital interplay between the host organism and its microbiota is initiated at birth and gradually changes until it stabilises during childhood and adulthood. Among the non-age-related factors that affect the host microbiota are internal factors, such as host genetics and the state of the immune system, and external factors, such as antibiotic usage and dietary habits [48].

The microbial content of the GIT depends on passage rates, host secretions, nutrient availability and the surrounding tissues. The microbial content ranges from a small number of microbes with low diversity in the stomach to a large number with wide diversity in the large intestine, in which the acidity decreases, which highly favours bacterial colonisation [49].

The gut microbiota has a profound influence on human health because of many direct and indirect interactions with its host. This microbial community is involved in many of the host’s nutritional, developmental and immunological functions, and contributes to the regulation of several metabolic pathways via symbiotic host-microbiome signalling systems [50]. The equilibrium in the microbiota ecosystem is critical for maintaining host homeostasis, whereas metabolic disorders are coupled to disturbance in the composition and/or functions of the gut microbiota [51].

Even prior to the advancement of research on the gut microbiota, probiotics (beneficial bacteria mostly belonging to the genera *Lactobacillus* and *Bifidobacterium*) were used in nutrition or in the manufacture of functional food products. The benefits of probiotics include the inhibition of a variety of pathogens, production of short-chain fatty acids (SCFAs), stimulation of the immune system and reinforcement of intestinal epithelial cell tight junctions [52].

Although over 50 phyla of gut microbes have been identified, most bacterial species belong to five phyla, namely, Firmicutes, Bacteroidetes, Actinobacteria, Proteobacteria and Verrucomicrobia, with Firmicutes and Bacteroidetes being the dominant phyla. Among the major genera classified under these phyla are *Clostridium/Clostridioides*, *Enterococcus*, *Lactobacillus*, and *Ruminococcus* (Firmicutes); *Bacteroides* and *Prevotella* (Bacteroidetes); *Bifidobacterium* (Actinobacteria); and *Escherichia* (Proteobacteria). Verrucomicrobia include *Akkermansia mucinophila,* which has a considerable influence on human health despite being relatively low in abundance [53].

#### 3.2.2. Metabolic Potential of the Gut Microbiota

The phylogenetic composition of the gut microbiota is thought to be unique and stable over time for each individual. A vast diversity of intestinal microbial species is observed among individuals, and this variability is a result of multiple factors, such as age, health, diet (including probiotics), antibiotic usage, and geographical location of the individual [54]. Consequently, each human has a unique gut microbiome fingerprint.

In addition to the phylogenetic structure of the microbiota, the collective intestinal microbial metagenome (i.e., the microbiome) is quite complex and has been estimated to contain more than five million genes, approximately 200 times more than the number of human genes [55]. Owing to the great diversity of intestinal bacterial species and the large number of genes harboured by these species, the metabolic capacity of the intestinal microbiome is estimated to be 100-fold greater than that of the human liver [56,57]. This remarkable coding capacity results in several biological activities that are known to benefit the host’s health. For example, gut bacteria encode enzymes involved in the formation of beneficial vitamins and metabolites [55]. These bacteria possess many biochemical pathways that humans have not developed, such as the bioactivation, degradation and metabolism of exogenous compounds, including indigestible dietary compounds [58]. Lately, the impact of the gut microbiota on drugs and the mutual drug–microbiome interactions have been highlighted, and the fields of pharmacomicrobiomics and toxicomicrobiomics are rapidly developing to systematically study such interactions and catalogue their pharmacotherapeutic and clinical impacts [59,60,61,62,63].

Gut microbial enzymes include β-glucosidase, β-glucuronidase, α-rhamnosidase, sulfatase, and esterases, which can hydrolyse glycosides, glucuronides, sulfates, amides and esters, respectively. Other reactions include aromatic ring cleavage, reduction (by reductases and hydrogenases), decarboxylation, demethylation, isomerisation, and dihydroxylation [64].

#### 3.2.3. Metabolic Effect of the Gut Microbiota on Tannins

Members of the gut microbiota ferment non-digestible xenobiotic-like procyanidins to produce bioavailable metabolites that exert pharmacological effects upon systematic absorption. Simultaneously, this fermentation process also leads to the generation of SCFAs that, in turn, reduce the pH of the gut lumen, facilitate the absorption of ions and act as direct sources of energy for epithelial cells [56]. Studies have indicated a high percentage of non-absorbable dietary polyphenols reaching the colon intact [65], and the largest proportion of ingested polyphenols is excreted in urine in the form of microbiota-mediated metabolites [66].

The absorption and bioavailability of polyphenols are largely influenced by the structures of these compounds. In terms of absorption, only aglycones and, to a lesser extent, a few glycosides can be absorbed in the intestinal mucosa. Thus, an essential step for the absorption of dietary polyphenols is the host- or microbiota-mediated release of the corresponding aglycone from the parent polyphenol precursor. This step, catalysed by microbial glucuronidases and sulfatases, allows for the reuptake of aglycones. The latter need to be further metabolised by the colonic microbiota into absorbable simple aromatic compounds [67].

##### Gut Microbiota-Mediated Metabolism of Hydrolysable Tannins

Under the influence of many bacterial enzymes, HTs are broken down into gallic acid, pyrogallol, and phloroglucinol and eventually to acetate and butyrate. The tannase enzyme, produced by different bacterial groups, has the ability to easily hydrolyse and degrade GTs, but the effect of this enzyme is restricted to the hydrolysis of galloyl residues on the hexahydroydiphenoyl moiety of ellagitannins [68]. The main difference between the bacterial hydrolysis of GTs and ellagitannins is that the former yield gallic acid and glucose, owing to the esterase and depsidase activities of bacterial enzymes, while the latter mostly undergo lactonisation to produce ellagic acid.

**Figure 3:**
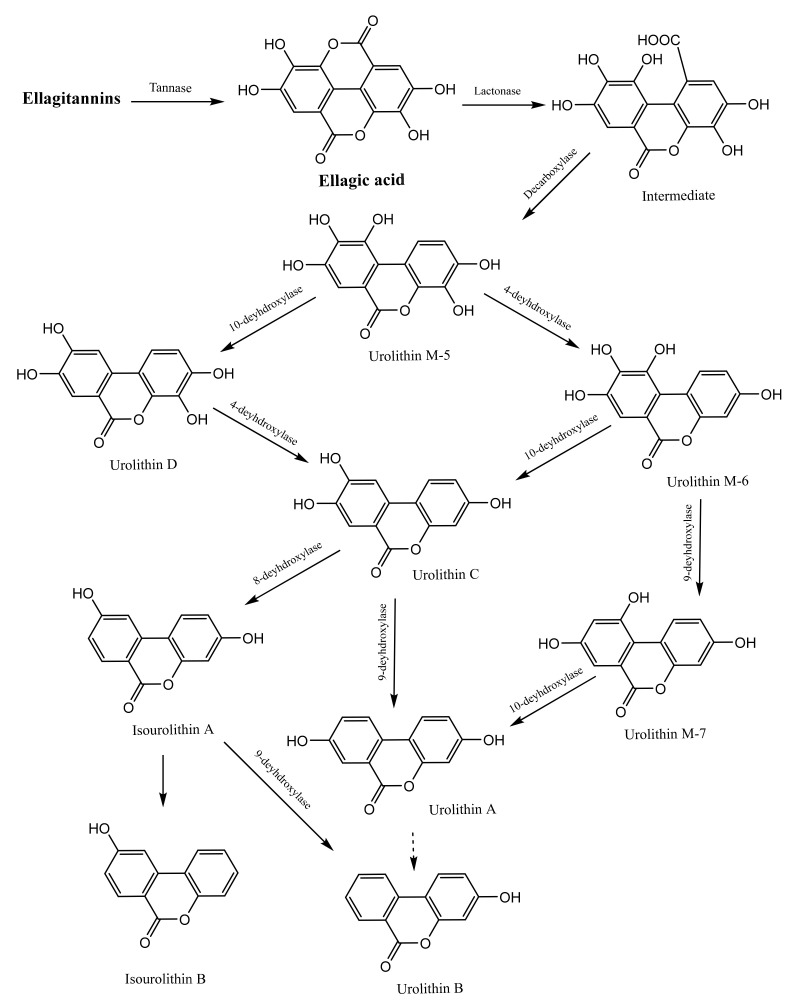
Microbiota-mediated biotransformation of ellagitannins to urolithins as detailed in [69].

Further bacterial metabolism of ellagic acid in the colon yields urolithin A and its mono-hydroxylated derivative, urolithin B. This bacterial transformation of ellagic acid is mediated by a series of lactone ring cleavage, decarboxylation and dehydroxylation reactions, starting with the formation of urolithin M-5 and ending with the formation of urolithin A and B (Figure 3). Bacterial hydrolysis of ellagitannins and ellagic acid is necessary for the absorption of these compounds, because these compounds exhibit moderate absorption, while urolithins can be easily absorbed [69].

All previous reports have indicated the bacterial origin of urolithins, and some studies have attempted to identify the specific bacterial species responsible for this biotransformation. *Butyrivibrio* spp. and lactobacilli with tannase activity, isolated from human faeces, were suggested among the bacterial species responsible for HT hydrolysis. Through the screening of 48 strains of *Bifidobacteria* and 1070 bacterial isolates from the faeces of a healthy woman, *Bifidobacterium pseudocatenulatum* INIA P815 was discovered as the only bacterial strain capable of producing urolithins A and B from ellagic acid [70]. Another species, *Gordonibacter urolithinfaciens*, was reported to metabolise ellagitannins into urolithins [71]. Members of a third bacterial genus, *Gordonibacter*, were found to transform ellagic acid into urolithins in healthy individuals who consumed ellagitannins in walnuts and pomegranate [72]. Urolithin A production was positively correlated with *Gordonibacter* abundance, which was also confirmed by in vitro studies [71,72,73,74]. The identification of such bacterial strains capable of producing pharmacologically active urolithins from ellagitannins may provide insight into the application of these strains in the development of probiotics and functional foods.

Another set of studies attempted to analyse the microbial metabolism of HTs. By feeding red raspberries containing ellagitannins to healthy volunteers and analysing the metabolites in their urine and plasma samples using UPLC-MS, Ludwig et al. [75] found that the plasma samples contained urolithins A and B, generated by the colonic bacterial metabolism of raspberry ellagitannins; however, these urolithins were excreted in urine as glucuronide and sulfate urolithin metabolites. Although the urinary recovery of urolithin metabolites was low (7%), the urolithins persisted in the circulatory system for a long duration. The bioavailability and possible protective effects of ellagitannins were attributed to their bacterial metabolism in the proximal and distal GIT [75].

In another study, several ellagitannin-containing herbs were incubated with slurries of human faecal samples, and the resulting metabolites were detected by UPLC-MS-MS. Urolithins A, B and C were detected in all the plant samples, as was the pure ellagitannin vescalagin standard [7]. Similarly, using UPLC-MS, Tulipani et al. [76] detected urolithins A, B, C and D in hydrolysed urine of humans who had consumed nut ellagitannins and urolithin-conjugated complexes in non-hydrolysed urine. Accordingly, they attributed the formation of such metabolites to extensive Phase II metabolism of nut ellagitannins [67]. In contrast, Truchado et al. [11] investigated whether processing ellagitannin–containing strawberries could affect the production and excretion of urolithins and thus whether the possible health effects of such metabolites could be accomplished through the administration of fresh and thermally processed strawberries to healthy individuals. Although thermal processing increased the level of free ellagic acid threefold, this processing had no effect on ellagic acid metabolism or subsequent urinary excretion of urolithin metabolites A and B as glucuronide. That study concluded that the rate or level of ellagic acid release from ellagitannins has no effect on the microbial transformation of this compound or any consequent health effects. Advances in analytics, mostly exemplified by ultra-performance liquid chromatography–mass spectroscopy (UPLC-MS) technology, led to most of the discoveries of mechanisms of gut tannin metabolism by the gut microbiota [11].

Similar results were obtained by Seeram et al. [10], who assessed urine and plasma samples from individuals given pomegranate juice containing the ellagitannin punicalagin. Ellagic acid and its dimethyl glucuronide derivative, in addition to urolithin A and B, were detected in all the analysed plasma and urine samples. The possible health effects of pomegranate juice have been attributed to the urolithin metabolites that persist in plasma and tissues for up to 21 days [10]. Furthermore, urolithins A and B and their glucuronide derivatives, as well as ellagic acid and its dimethyl glucuronide derivative, were detected by HPLC-MS-MS in all the plasma and urine samples of individuals who had consumed standardised pomegranate extracts. These metabolites had 32% higher antioxidant capacity than pomegranate extract [77]. Further studies demonstrated the possible inhibitory effects of urolithin metabolites on prostate cancer cells via inhibition of nuclear factor kappa-B activation in mice, which could be beneficial after initial treatment with radiation or surgery [78].

Although most of the reported studies agreed on the resulting metabolites of HT digestion, the residence times and quantities of these metabolites varied greatly among individuals. These variations may be attributed to variations in the composition of the colonic microbiota among individuals, dietary habits or health status.

##### Gut Microbiota-mediated Metabolism of Condensed Tannins

Flavan-3-ol compounds, e.g., epigallocatechin gallate (EGCG) from tea, are hydrolysed by salivary esterases to epigallocatechin [79]. The generated aglycones of monomer or dimer units are readily absorbed from the gut before reaching the colon. The generated gallic acid is further decarboxylated into pyrogallol [23]. Other factors affecting the bioavailability of flavan-3-ol in the gut are the interactions with proline-rich salivary proteins. This type of interaction is affected by the stereochemistry of the flavan-3-ol molecule or the monomer linkage pattern, as in the case of proanthocyanidin dimers [79].

**Figure 4:**
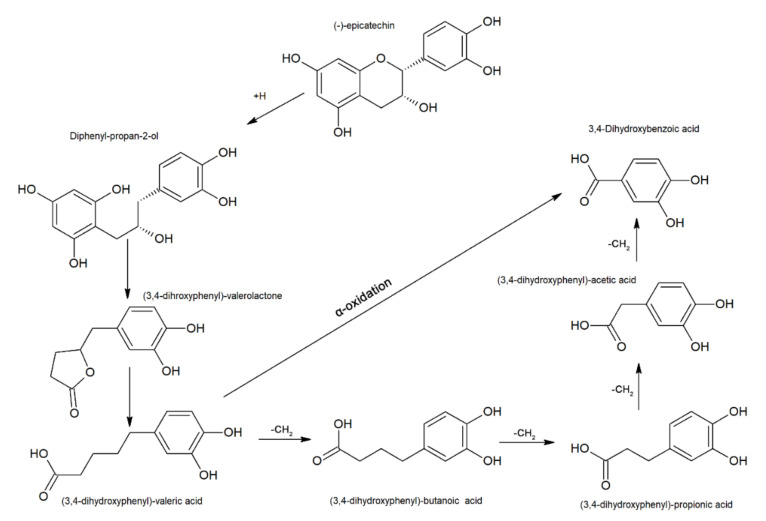
Gut microbial-mediated degradation pathway of flavan-3-ol.

In the colon, the flavan-3-ol monomer, polymers and associated bile-excreted Phase II metabolites are further metabolised, and the bioavailability of these compounds is highly dependent on their polymerisation [23]. The microbial degradation of monomeric flavan-3-ol units (Figure 4) involves C-ring fission to form diphenylpropan-2-ol via reductive cleavage. Ring fission results in the formation of metabolites belonging to one of the following categories:

(i) Five-carbon-ring fission-derived metabolites constitute the most abundant microbiota-mediated products. These products are formed by lactonisation of the diphenylpropan-2-ol unit, giving rise to hydroxyphenyl valerolactone derivatives such as 5(4′-hydroxyphenyl)-γ-valerolactone-3′-*O*-glucuronide, 5(4′-hydroxyphenyl)-γ-valerolactone-3′-sulfate, 5(3′-hydroxyphenyl)-γ-valerolactone-4′-*O*-glucuronide and glucuronide and sulfate conjugates of 5-phenyl-γ-valerolactone. Opening of the lactone ring results in the formation of 5-hydroxyphenyl-γ-valeric acid [43].

Three-, two- or one-carbon-ring fission-derived metabolites constitute a minor group of microbiota-mediated products. These products are formed by hydroxyphenyl valeric acid undergoing either successive β-oxidation, giving rise to hydroxyphenyl propionic acid derivatives followed by hydroxyl phenyl benzoic acid derivatives, or undergoing α-oxidation to yield hydroxyphenyl acetic acid directly. Along with the above reaction, dihydroxylation of A-ring hydroxyl groups can occur at Position 3 or 4, giving rise to 3- or 4-hydroxyphenyl derivatives, respectively [80]. Dehydroxylation and glycine conjugation subsequently give rise to hippuric acid, which is reported to be excreted in urine [43].

Despite the structural diversity between flavan-3-ol dimers A and B, these compounds release common microbial metabolites, such as 3-(3′,4′-dihydroxyphenyl)propionic acid (DHPA) and 3,4-dihydroxyphenylacetic acid (DHAA) [81]. 5-(3′, 4′-Dihydroxyphenyl)-γ-valerolactone (DHPV) and its conjugates have been recognised as the major microbial metabolites of procyanidins and (epi)catechins. After intake of various proanthocyanidin-rich foods, these compounds were detected in plasma and urine [82].

In contrast to previous research that indicated the α-oxidation reaction to occur in only dimer tannin units, α-oxidation catabolism was shown to affect both dimeric and flavan-3-ol monomers [15]. For example, DHAA and 3-hydroxyphenylacetic acid levels increased in the urine of rats after administration of procyanidin dimer B3 [83]. Other studies reported that after consumption of grape seed polyphenols, urinary excretion of 3-hydroxypropionic acid and 3-hydrophenylacetic acid increased significantly. Rios et al. [84] reported a significant increase in the urinary excretion of 3-hydroxyphenylpropionic acid, DHAA, 3-hydroxyphenylacetic acid, ferulic acid, vanillic acid, and 3-hydroxyl benzoic acid after administration of flavonol-rich chocolate to humans [84].

Depolymerisation of flavan-3-ol dimers to monomeric units has been reported for procyanidin B2 but only to a small extent (less than 10%). Other metabolites possibly derived from the A-ring of the top units of dimers have also been identified, including those derived via the formation of interflavan linkage (e.g., 5-(2,4-dihydroxyphenyl)-2-eno-valeric acid).

Likewise, in the case of monomeric flavan-3-ol, dimers were reported to undergo dehydroxylation at Position C3 or C4 [15]. Dehydroxylation of epigallocatechin occurs primarily at Position 5, with further dehydroxylation occurring at either Position 3 or 4 of the A-ring. However, the 3′,5′-dihydroxylated derivative of phenyl valerolactone has been identified, indicating dehydroxylation at Position 4 [85]. Various metabolites produced from CTs have been reported in the literature (Table 2).

Microbial metabolites (Table 2) either induce their pharmacological effects locally in the colon on microbiota cells and intestinal cells or are absorbed systematically into the blood stream, where these metabolites exert a systemic effect. However, studies investigating the effect of the microbiota on condensed tannin metabolism or relating variable pharmacological profiles to administered tannin regimes through the identification of definite metabotypes have multiple limitations. These limitations can be classified into subject-, sample-, study- and analysis-related limitations.

Most of the pioneering in vivo research on the effect of microbiota was performed in rats [86,87,88,89]. Although condensed tannin metabolism in rats was initially thought to resemble that in humans, subsequent research revealed distinctive variations. For example, in Phase II metabolism, sulfonation or glucuronidation are not prerequisites for flavan-3-ol methylation in rats, unlike in humans. This variation was evidenced by the isolation of 3′-*O*-methyl-epicatechin from rat plasma and urine [90]. Unmetabolised epicatechin was detected in the plasma and urine of rats but was absent in human counterpart samples. The same applies to the excretion of 5-(3′,4′-dihydroxyphenyl)-Ɣ-valerolactone in rat urine, with no further Phase II metabolism (glucuronidation, sulfation). The ring fission metabolite, 3′-hydroxyhippuric acid, is only excreted in human urine. These findings not only bring into question the relevance of previous studies on CTs and their metabolites, but also provide better insight into the appropriate selection and use of CTs and their metabolites in vitro or in vivo to induce certain pharmacological actions [43].

A caveat in most studies lies in the use of natural samples with poorly defined individual components, either because of poor purification or the unavailability of authentic substances under investigation in substantial amounts. This caveat typically leads to variable and sometimes contradictory results. The failure to account for matrix interference represents one reason for such contradictory results. More importantly, the lack of knowledge regarding sample composition leads to the inability to accurately determine structure–activity relationships (SARs) owing to the high diversity of flavan-3-ol structures and their multiple hydroxyl substitutions, stereochemistry with chiral carbon centres in ring C, esterification with gallic acid and polymerisation mode [91].

Taking into consideration that some flavan-3-ol metabolites are derived through endogenous metabolic pathways rather than flavan-3-ol uptake makes the interpretation of the concentrations of these metabolites, as products of tannin metabolism, increasingly complex. Hippuric acid and its derivatives are examples of such cases [43]. Research has shown that polymeric flavan-3-ol structures are more active than their monomer constituents, as in the case of the inhibition of DNA polymerase. The presence of a galloyl moiety at C-3 further enhanced the inhibitory effect of this compound against HeLa S3 cell proliferation. The same effect was proven by the synthesis of acetylated procyanidin B1 [91].

Most of the in vivo studies addressing the effect of the microbiota on condensed tannin metabolism measured the metabolic profile after acute ingestion of flavan-3-ol, which is not the case with for the normal population. Acute ingestion contributed to high inter-subject variability in the bioavailability of tannin metabolites and subsequently to high variability in pharmacological action [92]. Moreover, in vitro studies of condensed tannin fermentation with faeces-derived microbes did not consider the effect of Phase II metabolites generated in vivo.

The advancement of analytical techniques, such as hyphenated chromatography/high-resolution mass spectrometry, and the use of radiolabelled stable isotopes for the identification and quantification of condensed tannin metabolites have led to an improved understanding of the metabolite profiles and related metabolic pathways of subject samples. The use of multivariate analysis tools to characterise distinctive metabotypes of CTs has led to improved data analysis [92].

While research on flavones and HTs has revealed distinctive microbial metabotypes, this was not the case with CTs [91]. High inter-subject variability during in vivo studies, as well as the numerous degradation pathways, may have prevented the discovery of distinct metabolomic clusters. Moreover, these metabolites are released to variable extents according to host variability in terms of age, sex, race and dietary regime, which in turn leads to variability in the gut microbiome composition. Additional variability-generating factors are the chemical structures of ingested condensed, complexity of the food matrix, composition of the gut microbiota, and structures of co-ingested compounds that may affect bioavailability [93].

The diversity in metabolic reactions is also caused by the variability in microbes involved in the process. For example, *Eubacterium* spp., *Slackia equolifaciens, Slackia isoflavoniconvertens*, *Adlercreutzia equolifaciens,* and *Asaccharobacter celatus* were reported to cause fission of CTs via either ester cleavage or dehydroxylation of procyanidin dimers obtained from *Vaccinium macrocarpon* fruit extract (70% acetone), French maritime pine bark extract and purified procyanidin from *Salix caprea* leaf extracts [94]. The actinobacterial species *Eggerthella lenta* has been reported to cause ring C fission in flavan-3-ol monomers and further dehydroxylation at C-4 in the B-ring [95]. *Flavonifractor plautii* has been reported to generate hydroxyphenyl valerolactone and hydroxyphenyl valeric acid from the ring fission metabolite diaryl propan-2-ol [96,97]. Moreover, species that are involved in the hydrolysis (primary degradations) of tannins are *Eubacterium cellulosolvens*, *Bacteroides distasonis*, *Bacteroides ovatus, Bacteroides uniformis*, *Enterococcus casseliflavus*, *Eubacterium ramulus* and *Lachnospiraceae CG191* [98,99]. The aglycones and monomers resulting from the intial hydrolysis undergo further ring-cleavage and decarboxylation to form hydroxyphenyl propionic acid and hydroxyphenyl acetic acids [99,100]. Nutrikinetic studies have related the high values of polyphenol metabolites with members of the gut microbiota, e.g., with *Dialister*, *Prevotella,* and, to a lesser extent, *Anaerostipes* and *Turicibacter* [101].

A recent study tentatively identified three metabotype patterns based on biomarker metabolite abundance in urine upon prolonged administration of green tea extract and green coffee extract [91]. All these findings call for further research to elucidate how tannins affect the microbiota and which microbial strains are responsible for the different degradation pathways of tannins. Using large study populations and considering age, sex, health status, underlying disease and matrix interference during selection may help further elucidate such metabotypes, leading to an improved understanding of tannin-related health benefits at a molecular level. Additionally, most of the aforementioned biotransformed tannin products have yet to be assessed for their role in the gut microenvironment and for health-related or deleterious effects.

**Table 2:** Microbiota-mediated biotransformed tannin metabolites.

**Section 1: Metabolite Identification**						
**Category**	**Code**	**Name**	**Category**	**Code**	**Name**	
Valeric acid derivatives	1	(−)-5-(3′,4′,5′-Trihydroxyphenyl)-γ-valerolactone		36	3-Hydroxybenzoic acid sulfate	
	2	(−)-5-(3′,4′,5′-Trihydroxyphenyl)-γ-valerolactone glucuronide		37	4-Hydroxybenzoic acid sulfate	
	3	(−)-5-(3′,4′,5′-Trihydroxyphenyl)-γ-valerolactone sulfate	Procyanidin monomers metabolites/conjugates	38	(+)-Catechin	
	4	(−)-5-(3′,4′Dihydroxyphenyl)-γ-valerolactone		39	(−)-Epicatechin	
	5	(−)-5-(3′,4′Dihydroxyphenyl)-γ-valerolactone glucuronide		40	(–)-Epicatechin glucuronide	
	6	(−)-5-(3′,4′Dihydroxyphenyl)-γ-valerolactone methyl glucuronide		41	(-)-Epicatechin sulfate	
	7	(−)-5-(3′,4′Dihydroxyphenyl)-γ-valerolactone sulfate		42	(−)-Epicatechin-5/7-*O* -sulfate	
	8	5-(3′-Hydroxy phenyl)-γ-valerolactone		43	5/7-*O* -Sulfate-(−)-epicatechin-glucuronide	
	9	5-Hydroxyphenyl-γ-valerolactone-*O*-glucuronide		44	3′-*O* -Methyl-epicatechin	
	10	5-(3′-Hydroxyphenyl)-γ-valerolactone-4′-*O* –sulfate		45	4′-*O* -Methyl-epicatechin	
	11	5-(3′,4′-Dihydroxy phenyl)valeric acid		46	3′-*O* -Methyl-(−)-epicatechin-5/7-*O* -sulfate	
	12	5-(3′,4′-Dihydroxy phenyl)valeric acid-*O* -sulfate		47	4′-*O* -methyl-(–)-epicatechin-3′-*O* -beta-glucuronide	
	13	5-(3′-Hydroxy phenyl)valeric acid		48	(–)-Epigallocatechin glucuronide	
	14	4-Hydroxy-5-(3′,4′-dihydroxyphenyl)valeric acid		49	Methylated epigallocatechin glucuronide	
	15	4-Hydroxy-5-(3′,4′-dihydroxyphenyl)valeric acid-*O* -sulfate		50	Methylated epigallocatechin sulfate	
	16	3-*O* -Methyl-4-hydroxy-5-(3′,4′-dihydroxyphenyl)valeric acid-*O* –sulfate	Procyanidin dimers and other polyphenol metabolites/conjugates	51	Procyanidin dimers metabolites *	
Propionic acid metabolites/conjugates	17	3,4-Dihydroxyphenyl propan-2-ol		52	Procyanidin dimers metabolites **	
	18	3,4-Dihydroxyphenyl propan-2-ol. Dihydrate		53	Vanillic acid	
	19	3,4-Dihydroxyphenyl propan-2-ol-*O*-glucuronide		54	Homovanillic acid	
	20	1-(3′,4′-Dihydroxyphenyl)-3-(2′′,4′′,6′′- trihydroxyphenyl)propan-2-ol		55	Homovanillyl alcohol	
	21	3-(3′,4′-Dihydroxy phenyl)propionic acid		56	Gallic acid	
	22	3-(3,4-Dihytdroxyphenyl)propionic acid sulfate		57	3-O -Methyl gallic acid	
	23	3-(3′-Hydroxy phenyl)propionic acid		58	M-Coumaric sulfate	
	24	3-(3′-Hydroxy phenyl)propionic acid sulfate		59	*p*-Coumaric sulfate	
	25	3-(4-Hytdroxyphenyl)propionic acid		60	Ferulic acid sulfate	
	26	3-(4-Hydroxyphenyl)propionic acid sulfate		61	3-*O* -Protocatechuic acid sulfate	
	27	3-Phenylpropionic acid	Hydrolysable tannins metabolites/conjugates	62	Urolithin A	
Acetic and benzoic acid metabolites/conjugates	28	2-(3,4-Dihydroxyphenyl)acetic acid		63	Hydroxyl urolithin A	
	29	2-(3,4-Dihydroxyphenyl)acetic acid sulfate		64	Urolithin A glucuronide	
	30	2-(3′-Hydroxyphenyl)acetic acid		65	Urolithin B	
	31	2-(3′-Hydroxyphenyl)acetic acid sulfate		66	Urolithin B glucuronide	
	32	2-(4′-Hydroxyphenyl)acetic acid		67	Urolithin C	
	33	2-Phenylacetic acid		68	Urolithin D	
	34	Benzoic acid		69	Ellagic acid	
	35	3-Hydroxybenzoic acid		70	Dimethylellagic acid glucuronide	
**Section 2: Material, detection and reference**						
**Source**			**Metabolite code**	**Detected in**	**Detection mode**	**Reference**
*Filipendula ulmaria*, *Geranium pratense*, *Geranium robertianum*, *Geum urbanum* root and rhizome, *Lythrum salicaria*, *Potentilla anserina*, *Potentilla erecta* rhizome, *Quercus robur*, *Rubus idaeus* leaf, *Rubus fruticosus* L. and pure ellagitannin vescalagin			62-65-67 ***	Fermentation with human microbiota	LC-MS	[7]
Punicalagin			62-65	Fermentation with human microbiota	LC-MS	[19]
Red raspberries (*Rubus idaeus* L.)			62-65	Urine (human)	LC-MS	[75]
Cocoa powder			4-28-30	Urine (human)	LC-MS	[80]
Proanthocyanidin dimers			4-8-13-20-21-23-28-30-32	Fermentation with human microbiota		[81]
Green tea			2-3-40-48-49-50	Urine (human)	LC-MS	[85]
(-)-epicatechin			4	Urine (rats)	LC-ECD	[86]
EC, PC B1 and Polymeric PC fraction of cocoa			4-14-32-44-45-51	Urine/plasma (human)	LC-MS, GC-MS	[102]
Epicatechin, catechin, procyanidin B2			4-8-15-23-27-28-30-32-33-34	Fermentation with human microbiota	GC-MS	[103]
Grape seed proanthocyanidin extract			4-21-23-26-27-28-30-32-33-34-35-38-39-53-54-55-56-57	Plasma (rats)	LC-MS	[87]
Procyanidin B2, Epicatechin			4-8-11-14-15-20-21-23-30-33-39	Fermentation with human microbiota		[104]
Apple polyphenol extract			5-6-12-14-21	Urine/plasma (human)		[105]
Procyanidin B2			7-15-16-17-18-19-22-24-29-31-36-37-41-42-43-46-51-58-59-60-61	Urine (rats)	LC-DAD-MS	[88]
Partially purified apple procyanidin (PPCP)			9-11-15-47	Plasma (rats)	LC-MS, NMR	[106]
Carnberry juice			10	Plasma (human)	LC-MS	[107]
Pomegranate and walnuts			62-65	Urine/plasma/feces (human)	LC-MS	[108]
Strawberries, red raspberries, walnuts and oak-aged red wine.			62-65	Urine (human)	LC-MS	[12]
Pomegranate juice			62-65-69-70	Urine/plasma (human)	LC-MS	[8]
			62-63-64-65-70	Plasma (human)	LC-MS	[77]
Strawberry			62-64-65-66	Urine (human)	LC-MS	[11]
Walnuts (*Juglans regia* L.), hazelnuts (*Corylus avellana* L.), and almonds (*Prunus dulcis* Mill.)			62-65-67-68	Urine (human)	LC-MS	[76]

* 3′-O-Methyl upper procyanidin B2, 3′-O-Methyl upper-3′/4′-methyl lower procyanidin B2, 3′-O -Methyl upper-3′/4′-methyl lower procyanidin B2.2H_2_O, 3′/4′-O-Methyl lower procyanidin B2, 3′/4′-O-Methyl lower procyanidin B2.(H_2_O) sulfate, 3′-O-Methyl upper-3′/4′-methyl lower procyanidin B2 glucuronide, 3′-O-Methyl upper-3′/4′-methyl-lower procyanidin B2(H_2_O) sulfate, 4′-O-Methyl upper procyanidin B2, 6/8-Hydroxy upper procyanidin B2 sulfate, Hydrogenation procyanidin B2, Methyl C-ring cleavage procyanidin B2, Procyanidin B2 sulfate. ** Methyl-O-Procyanidin B1. *** Dashes (-) between numbers in Section 2 of the table do not indicate ranges.

#### 3.2.4. Gut Metabotypes and Tannin Metabolism

Many studies have reported high inter-individual variability in tannin metabolism [109,110,111]. Such variation calls for the development of personalised treatment and targeted nutrition strategies based on individual metabotype profiles. A gut metabotype refers to the phenotype associated with metabolism by the gut microbiota of a parent compound (such as a polyphenol compound) to specific metabolites [112,113]. In general, the production and detection of specific microbial metabolites could serve as biomarkers for specific gut microbial communities [114]. In the case of tannins, gut metabotypes are strongly linked to the production of specific microbiota-associated metabolites from several tannins, including proanthocyanidins [115] and ellagitannins [116,117], and hence many health benefits are associated with tannin intake. For example, the production of 4-hydroxyphenylacetate, one of the microbiota-associated metabolites of red wine proanthocyanidins, is the main biomarker used in clinical phenotype-based clustering of patients with cardiovascular risk according to their different responses to red wine intake [5].

As previously mentioned, urolithins, which are dibenzo(*b*,*d*)pyran-6-one derivatives, are produced by the human gut microbial biotransformation of ellagitannins and ellagic acid. These metabolites have been suggested to be responsible for the pharmacological actions of ellagitannin and ellagic acid and the associated health benefits [60]. Three major ellagitannin metabotypes (A, B and 0) have been described [116]. Metabotype A is characterised by the production of urolithin A and its related conjugates. The production of urolithin B and/or isourolithin A, in addition to urolithin A production, is the main biomarker that characterises metabotype B, while metabotype 0 is characterised by the lack of production of any of the aforementioned urolithins (only urolithin M5 and urolithin M6 have been found in this metabotype) [117].

Evidently, such inter-individual variability in urolithin metabotypes is associated with differences in the gut microbial composition. For example, the presence of *Gordonibacter* is associated with urolithin A production in vivo [72]. Higher levels of *Gordonibacter* were found in individuals with metabotype A than in those with metabotype B [118]. Interestingly, metabotype B was reported to be dominant in subjects with colorectal cancer and metabolic syndrome, as well as overweight subjects. This pattern suggests a correlation between metabotype B and gut dysbiosis, and hence suggests negative health effects [117]. The association among urolithin metabotypes, gut dysbiosis and health status is being scrutinised by the scientific community [72,116,119].

Recently, an ellagitannin-rich pomegranate extract was found to selectively improve cardio-metabolic biomarkers in individuals with urolithin metabotype B [120]. Moreover, in a placebo-controlled, randomised, clinical trial, administration of ellagitannin-rich pomegranate extract did not have significant effects on cardiovascular risk markers in volunteers. However, in the same study, a significant improvement in cardiovascular risk markers (such as total cholesterol, LDL-cholesterol, apolipoprotein B and oxidised-LDL-cholesterol) was only reported in individuals with metabotype B, when the researchers stratified the volunteers into groups according to urolithin metabotype [120].

In conclusion, comprehensive knowledge and understanding of the metabolic fate of dietary tannins, microbiota–tannin interactions and tannin-metabolism-associated phenotypes (metabotypes) is of great importance, allowing researchers to harness these effects and improve the benefits of dietary tannins and polyphenols.

## 4. Biological Effects of Gut-Biotransformed Metabolites of Tannins

### 4.1. Biological Effects of Gut-Biotransformed Metabolites of CTs

Along with the research to identify tannin metabolites and determine their absorption, distribution, metabolism and excretion (ADME), another line of research has been dedicated to identifying the biological effects of tannin metabolites. These lines of research are complementary to each other and correlate the bioavailability of certain metabolites to biological action, allowing for the assignment of these identified biological actions to their causative compounds in terms of both target location and effective concentration (metabolite concentration at target organ). The following sections specifically highlight the biological effects of microbiota-mediated tannin metabolites.

#### 4.1.1. Chemopreventive Activities of CT Metabolites

The US National Cancer Institute (NCI) defines chemoprevention as the use of drugs, vitamins or other agents to reduce the risk or delay the development or recurrence of cancer [121]. However, a chemopreventive agent is not necessarily able to treat cancer. Several modes are associated with chemoprevention. These include inhibition of oxidative stress and related damage, modulation of cellular signalling in inflammatory response, induction of cellular Phase II detoxifying/antioxidant genes, activation of liver metabolic enzymes responsible for the detoxification of carcinogens, anti-inflammatory effects, blockage of metabolic activation in cancer cells, blockage of the binding of DNA to carcinogens, repair of the DNA damage-mediated induction of apoptosis in precancerous or malignant cells, inhibition of tumour cell growth and metastasis and, lastly, exertion of antiangiogenic effects [122,123].

Mechanisms of chemoprevention include perturbation of tumours at various stages of initiation, promotion, or progression or throughout the carcinogenic process [124]. The chemopreventive effects of phytochemicals usually involve combinations of the abovementioned mechanisms rather than only one [123]. Clinical pre-trials have indicated procyanidins as promising candidates in cancer prevention and/or treatment. These compounds may be used either alone or as adjunct therapies with other chemotherapeutic regimens [125].

Generally, studies addressing the chemopreventive action of procyanidins have mainly performed preclinical ex vivo or in vivo experiments on animal models, normal or cancer cell lines, with a few subsequent attempts to proceed into clinical trials. These studies involved investigation of the effect of either flavan-3-ol monomers, oligomers (with special attention to B type), polymers or CT metabolites [125]. Unlike proanthocyanidins, flavan-3-ol metabolites had no marked effects as chemopreventive agents. Lambert et al. [126] reported inhibition of intestinal epithelial cancer cells INT-407 by 5-(3′,4′,5′-trihydroxyphenyl)-γ-valerolactone. 5-(3′,4′,5′-Trihydroxyphenyl)-γ-valeric acid, 5-(3′,4′-dihydroxyphenyl)-γ-valeric acid, and 4-hydroxy-5-(3′,4′,5′-trihydroxyphenyl)-γ-valeric acid inhibited the proliferation of human cervical cancer HeLa cells [127]. Moreover, tea catechin metabolites were shown to prevent cancer through immune stimulation. The green tea catechin metabolite 5-(3′,5′-dihydroxyphenyl)-γ-valerolactone enhanced the activity of CD4^+^ T cells as well as natural cytotoxic cell activity in vivo. According to the study, this effect was attributed to the 4-hydroxyl group in the B-ring of the metabolite [128].

#### 4.1.2. Other Biological Activities of CT Gut-biotransformed Metabolites

The biological activity of 5-(3′,4′-dihydroxyphenyl)-γ-valerolactone and 5-(3′-methoxy-4′-hydroxyphenyl)-γ-valerolactone is higher than that of their parent flavan-3-ol compound (+)-catechin or the maritime pine bark extract. These compounds exert antioxidant activity by inhibiting the metalloproteinases MMP-1, MMP-2 and MMP-9 after oral administration of pycnogenol. Another metabolite, 5-(3′,4′-dihydroxyphenyl)-γ-valerolactone, exhibits superior oxygen radical scavenging activity compared to the antioxidants (+)-catechin, ascorbic acid, and trolox [129]. Further research on this subject revealed the role of 5-(3′,4′-dihydroxyphenyl)-γ-valerolactone in the inhibition of NO production through inhibition of inducible NO synthase (iNOS) in vivo in a concentration-dependent manner and demonstrated the accumulation of the metabolite in target cells, further explaining the in vivo activity at concentration ranges lower than the in vitro experimental concentration used to induce the antioxidant effect [130].

Takagaki and Nanjo [115] demonstrated the effect of the gallocatechin and epigallocatechin ring fission metabolites, which act as angiotensin-converting enzyme inhibitors, in the reduction in systolic blood pressure in rats [115]. The hydroxyphenyl valeric acid metabolites were ranked according to decreasing efficiency as follows: 5-(3′,4′,5′-trihydroxyphenyl)-γ-valerolactone, trihydroxyphenyl 4-hydroxyvaleric acid, dihydroxyphenyl 4-hydroxyvaleric acid, and 5-(3′,5′-dihydroxyphenyl)-γ-valerolactone. When 5-(3′,4′,5′-trihydroxyphenyl)-γ-valerolactone and 5-(3′,5′-dihydroxyphenyl)-γ-valerolactone were orally fed to spontaneous hypertensive rats (SHR), these compounds reduced systolic blood pressure within 1 and 4 h, respectively, indicating their hypotensive effects in vivo. Furthermore Adnan et al. [131] demonstrated that hypertension may be induced in normotensive strains by modification of the gut microbiota by introducing the microbiome of SHR into normotensive rat strains. However, contradictory results were obtained, as hydroxyphenyl valerolactone derivatives showed no vasorelaxant activity upon testing on mouse arteries in vitro [132].

In recent studies, 5-(3′,4′-dihydroxyphenyl)-γ-valerolactone exhibited anti-atherosclerotic effects by preventing the effect of the TNF-α-induced adhesion of human monocyte cells to venous endothelial cells. Inhibition of TNF-α reduces the expression of vascular cell adhesion molecule-1 and monocyte chemotactic protein-1, which are biomarkers of atherosclerosis. TNF-α inhibition inhibits the activation of nuclear factor kappa-B transcription and phosphorylation of I kappa-B kinase and IκBα [133]. Mele et al. [134] identified the role of (R)-5-(3′,4′-dihydroxyphenyl)-γ-valerolactone and (R)-5-(3′-hydroxyphenyl)-γ-valerolactone-4′-*O*-sulfate in the protection of brown adipocytes from increased hydrogen peroxide levels, which induces reactive oxygen species production [134].

Peron et al. [135] attributed the antiadhesive effect on uropathogenic *Escherichia coli* to the excretion of the microbiota-mediated metabolites valeric acid and valerolactone derivatives in urine samples 6 and 8 h after oral administration of cranberries (major source of PAC-A) [135].

Several epidemiological studies identified a relationship between the administration of flavan-3-ols and a lower risk of neurodegenerative disorders, such as dementia and Alzheimer’s disease. Consistent with these studies, Unno et al. [136] demonstrated that 5-(3′,5′-dihydroxyphenyl)-γ-valerolactone, pyrogallol, and their glucuronide and sulfate conjugates, which are microbial metabolites of epigallocatechin, the main flavan-3-ol in tea, cross the blood–brain barrier and have an effect on neurite proliferation and longevity in vitro [136]. Neurodegenerative disorders, such as Alzheimer’s disease, are characterised by the cerebral disposition of amyloid beta protein plaques. Studies showed that EGCG and gallic acid reduce beta amyloid disposition [137].

**Figure 5:**
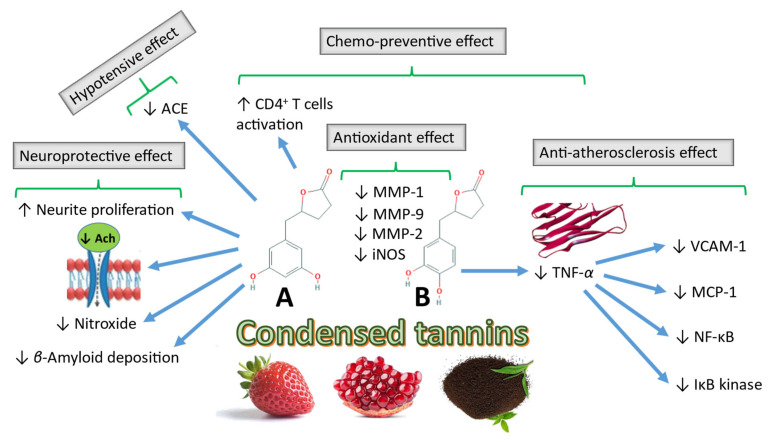
Examples of the reported biological activities of bacterial metabolites of CTs and their proposed mechanisms of action: (**A**) 5-(3′,5′-dihydroxyphenyl)-γ-valerolactone; (**B**) 5-(3′,4′-dihydroxyphenyl)-γ-valerolactone. Plants in the bottom are (left-to-right): strawberry, pomegranate, tea.

An ex vivo study run on the nematode *Caenorhabditis elegans* showed that pomegranate puniclalgan-derived urolithins exhibited a protective effect against amyloid β1-42–induced neurotoxicity and paralysis [138]. An in vivo study indicated that rapid decline in the nitroxide, 3-methoxycarbonyl-2,2,5,5-tetramethylpyrrolidine-1-oxyl (PCAM), may be attributed to higher brain antioxidant ability in mice fed a diet containing mimosa tannin (MMT), compared to other mice fed a normal rodent diet [139]. Further research is needed to assess the effects of metabolites obtained from other flavan-3-ol sources, such as grape, and to explore the bioavailability in humans, compared to that in rats [140]. Figure 5 illustrates some of the reported biological activities of CTs microbial metabolites and their proposed mechanisms of action.

### 4.2. Biological Effects of Gut-Biotransformed HT Metabolites

Owing to their higher bioavailability than ellagitannins, urolithins have been extensively studied for their possible pharmacological effects and their potential mechanism of action. Such effects mostly include their anti-inflammatory, antioxidant, anticancer, anti-atherosclerotic and cardioprotective effects.

#### 4.2.1. Anti-Inflammatory and Antioxidant Activities of Urolithins

The anti-inflammatory effect of urolithins is among the most extensively studied effects of these bacterial-derived metabolites. Several studies investigated the effect of urolithins on various inflammatory markers, attributing the anti-inflammatory activity of ellagitannin-containing plants to these metabolites rather than to the poorly bioavailable ellagitannins. As exemplified in the work of Boakye et al. [141], urolithin A was identified as being a stronger inhibitor of M1 macrophage polarisation than the ellagitannin geraniin. This inhibition led to a significant reduction in inflammatory marker production. Additionally, urolithin A markedly inhibited pro-inflammatory gene expression, with a subsequent reduction in the nuclear abundance of p65 (nuclear factor-κB; NF-κB) and M1 (LPS) polarisation. Urolithin A interfered with Akt/mTOR (mammalian target of rapamycin) signalling, suggesting that the intestinal microbiota-mediated metabolism of geraniin to urolithin may boost the anti-inflammatory effect of this compound.

Similar results were obtained by Komatsu et al. [142]. Having investigated the anti-inflammatory effect of urolithin A on lipopolysaccharide (LPS)-stimulated RAW264 macrophages, they found that Urolithin A inhibited NF-κB and activator protein-1 (AP-1) activation as well as the phosphorylation of Akt and c-Jun N-terminal kinase (JNK), resulting in the suppression of pro-inflammatory mediator production [142]. In another study, the anti-inflammatory and antioxidant activities of urolithin B were investigated against activated microglia in an attempt to elucidate the underlying molecular mechanisms [143]. Urolithin B inhibited the production of NO, TNF-α and pro-inflammatory cytokines but increased the production of the anti-inflammatory cytokine IL-10 [143]. Furthermore, in the aforementioned study by Piwowarski et al., the anti-inflammatory activity of urolithin metabolites was determined in a THP-1 cell line-derived macrophage model [7].

#### 4.2.2. Anticancer Activity of Urolithins

Like many other tannin metabolites, urolithins were tested for anticancer activity against various cancer types. For example, a mixture containing urolithin A and C showed the highest inhibitory activity against colon cancer stem cells and inhibited aldehyde dehydrogenase (ALDH) activity, considered to be a marker of chemoresistance. These results suggested a beneficial role of such metabolites in the prevention of chemoresistance and relapse of colon cancer [144]. Moreover, Zhou et al. [145] assessed the effect of the anticancer activity of methylated urolithin A on the viability of human prostate cancer cells (DU145). Strong inhibition of microRNA 21 (miR-21), responsible for the stimulation of cell invasion and metastasis in cancer cells, was observed in vitro and in vivo with subsequent inhibition of the tumour suppressor gene phosphatase and tensin homologue (PTEN), indicating potential anticancer activity against prostate cancer [145].

#### 4.2.3. Anti-Atherosclerotic and Cardioprotective Activities of Urolithins

**Figure 6:**
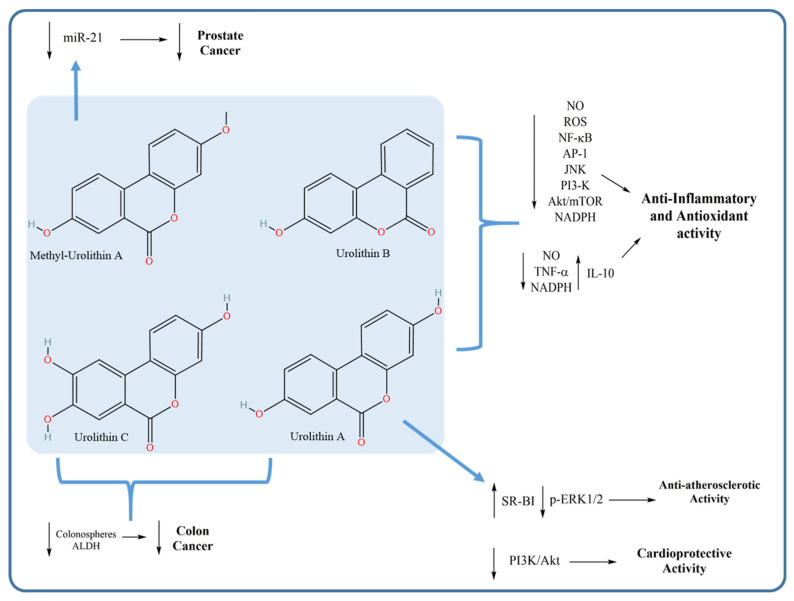
Reported action mechanisms of tannin-derived urolithins, highlighted in blue, with their related pharmacological activities.

Cardioprotection is another important bioactivity of urolithins. A potential therapeutic effect of urolithin A was demonstrated in atherosclerosis via attenuation of atherosclerotic lesions in Wistar rats [146]. Additionally, the role of urolithin A in the alleviation of myocardial ischaemia/reperfusion injury in myocardial cells was investigated in vitro and in vivo [147]; its cardioprotective activity was found to be mediated via the inhibition of the phosphatidylinositol 3-kinase (PI3K)/Akt pathway [147]. Figure 6 summarises the reported mechanisms of action of urolithins with their related pharmacological activities.

Taken together, most of the studies on the biological effects of tannin metabolites were performed in vitro, and relatively few in vivo studies have been conducted. Differences in the active concentrations of metabolites applied in vitro compared to the active concentrations in vivo led to some contradictory results. Greater attention should be paid to the use of isolated precursors in in vivo trials to avoid misinterpretation of biological action due to the concerted effect of various metabolites. Finally, the deposition of such metabolites in target organs should be identified to avoid misinterpretation of the in vivo active concentration compared to the in vitro active concentration.

### 4.3. Mining the Correlation between the Chemical Space of Gut-Biotransformed Tannin Metabolites and Biological Effects

Discovering the molecular signatures of gut-biotransformed tannin metabolites that are correlated with chemical and biological activity would help to reveal structural features of new unknown metabolites or compounds, and move pharmacomicrobiomic research from observation to prediction. ML algorithms have been widely used in predicting chemical, biological and physical characteristics of compounds. Pharma companies consider ML a potential tool to help facilitate drug research, lower the cost of drug development and enhance efficiency. To this end, we aimed to discover in silico the most important structure features of different gut-biotransformed tannin metabolites by developing four predictive models to determine whether they are antioxidant, anti-inflammatory, anticancer or anti-atherosclerotic, and by using Deepchem algorithms [148] along with Lime library for XAI [149].

**Figure 7:**
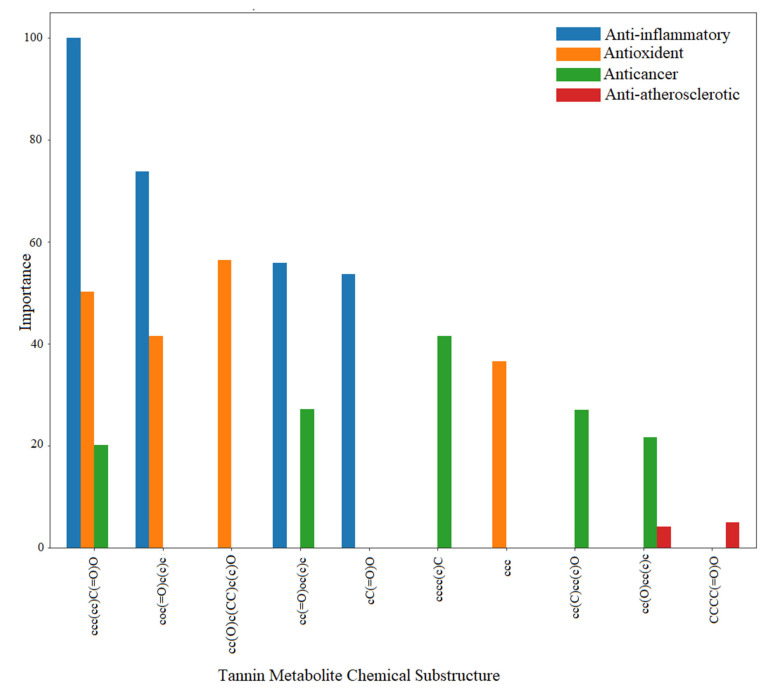
Importance of tannin metabolite chemical substructures vs. biological effects.

Our data of compounds and metabolites were extracted from the FoodDB database [150], and they consist of 528 simplified molecular-input line-entry system (SMILE) structures, distributed as follows: 251 for antioxident, 67 for anti-cancer, 23 for anti-atherosclerotic and 187 for anti-inflammatory activities. The cardioprotective activity was dropped out because of insufficiency of data to properly train a predictive model. The performance of our models for unseen metabolites ranged from 83% to 88% of the area under the receiver operating characteristic curve (ROC-AUC) score, which represents how capable the classification model is at distinguishing between positive and negative bioactivity. Tables S1 and S2 detail ROC-AUC-scores per biological effects and the deep neural network architecture, respectively. At the end of the process, metabolite substructures yielded by our predictive models were shortlisted based on ‘importance’ (Figure 7 and Table 3).

**Figure 8:**
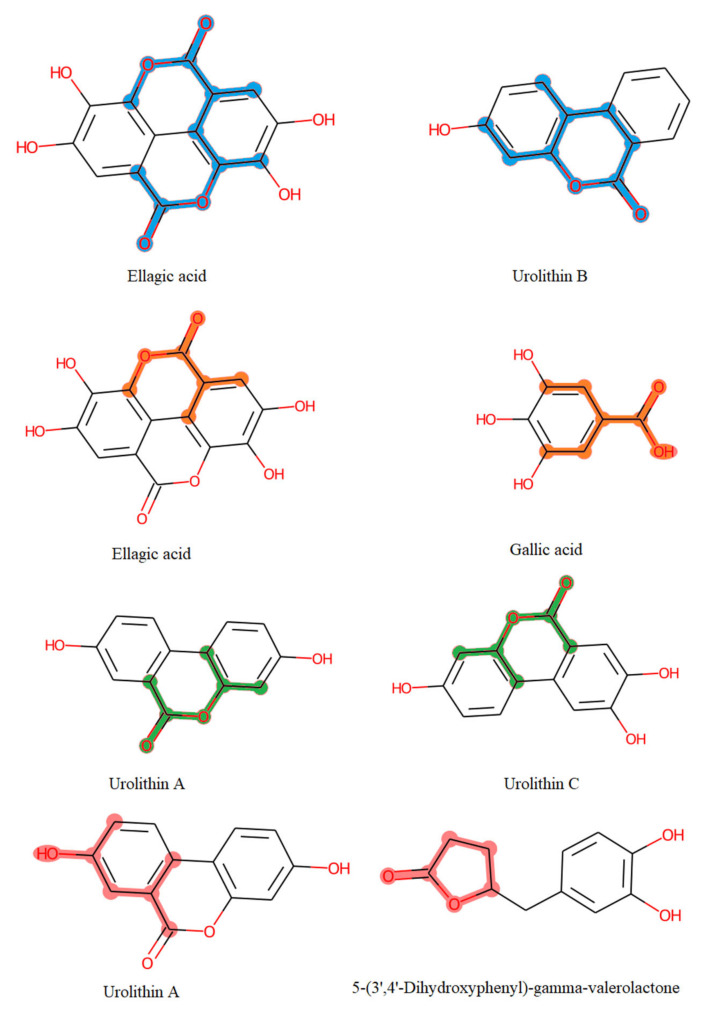
Examples of chemical motifs indicating biological effects. Blue: ani-inflammatory; orange: antioxidant; green: anticancer; red: anti-atherosclerotic. Coloured motifs represent structural positive contributions towards health effect.

Importance here denotes the weight of a chemical substructure predicted by a model divided by the sum of weights of all important chemical substructures. For example, the chemical substructure “ccc(cc)C(=O)O” is important for three biological effects (anti-inflammatory, antioxidant and anticancer) and was found in both gallic acid and vanillic acid metabolites, while the motif “coc(=O)c(c)c” is related to anti-inflammatory and antioxidant health effects and was derived from Ellagic acid metabolite (Figure 7 and Table 3). Urolithin A, B and C metabolites share the chemical motif “cc(=O)oc(c)c”, which is linked to anti-inflammatory and anticancer effects. Figure 8, Figures S1–S4 illustrate examples of correctly predicted chemical motifs by our predictive models.

## 5. Conclusions and Future Directions

Tannins are ubiquitous in most plant foods and beverages, and most of the reported health benefits of these products are due to tannins. Interestingly, the ability of tannins to exert such health effects mainly depends on the bioavailability of these compounds, which varies greatly among different sites of the gastrointestinal tract. The microbial metabolism of tannins yields highly bioaccessible microbial metabolites that account for most of the systemic effects of tannins. Thus, the isolation of these key biotransformed metabolites may lead to the identification of novel biologically active natural products and lead compounds or to an improved understanding of tannin metabolism.

All these findings indicate the importance of further research to elucidate the mechanism by which the colonic microbiota affects different tannin classes, and to identify the microbial species/strains responsible for different degradation pathways. Most of the reported studies have the limitation of investigating the microbiota effects on tannin metabolism, or related variable pharmacological profiles to administered tannin regimes, through identification of definite metabotypes. Other limitations can be categorised as subject-, sample-, study- and analysis-related limitations.

In vivo studies are especially important because they better simulate the colonic environment, which favours the growth of anaerobic microbes. Such studies, however, are complicated by the non-culturable nature of most members of the colonic microbiota. The current review provides updated information regarding gut microbiota-mediated activation or biotransformation of HTs and CTs found in typical food sources. Understanding the metabolic fate of dietary tannins, tannin–microbiota interactions and tannin-metabolism-related phenotypes (metabotypes) is of great importance to better harness these effects for improved health benefits of dietary tannins and polyphenols and to identify metabolic biomarkers that may predict the fate and thus the bioactivity of these tannins.

Research on HTs, in particular, has revealed distinctive metabotypes, which are not as distinctive as those identified for CTs. There have been advances in the metabolite analysis technologies investigating the gut microbiota-mediated metabolism of tannins at high levels. Metabolomics, including both targeted and global metabolite-profiling strategies, is rapidly becoming the approach of choice across a broad range of sciences, including systems biology, drug discovery, molecular and cell biology, and other medical and agricultural sciences. New analytical and bioinformatic technologies are continually being developed or optimised, significantly increasing the cross-disciplinary capabilities of this new biological tool.

The metabolomes of gut organisms are quite complex, and metabolomic tools are being established to provide detailed insight into the biochemical compositions of living organisms. With recent developments in metabolomic techniques, it is now possible to detect several hundreds of metabolites simultaneously, and to compare samples reliably for differences and similarities in a semi-automated and essentially untargeted manner. Such a tool is of increasing value for analysis of crude biological extracts, often complicated by a high-background matrix, as in the case of analysis of microbiota-mediated metabolism.

In parallel to the tremendous advances in metabolomics, unprecedented breakthroughs in DNA sequencing technologies have also made the identification and functional profiling of complex microbial communities achievable, regardless of whether members of these communities can be cultured or isolated. In less than a decade, the Human Microbiome Project has successfully created a blueprint for major microbial taxa residing in or on human membranes, including the gastrointestinal tract, and DNA sequences of these microbes and their genes are added to public databases daily, allowing researchers to better understand the role of these resident bacteria in health and in the metabolism of food components, such as tannins [151,152]. The decoding of genomic and metagenomic data, along with metabolomics, through automated computational annotation [153,154,155] allows for the rapid identification of potential functional genes involved in food metabolism or dietary product alterations in the gut, which may lead to carcinogen deactivation or potentiate the effects of anti-carcinogenic agents. The only challenge that we foresee for pursuing such research is that it requires a combination of various skills and different experimental backgrounds.

## Figures and Tables

**Table 1:** Plants enriched in tannins and their class types.

Plant Name	Type of Tannins		Reference
**Hydrolysable Tannins**			
Pomegranate (*Punica granatum*)	Punicalagin	Ellagitannins	[10]
	Casuarictin		
	Pedunculagin		
Strawberry (*Fragaria ananassa*)	Sanguiin		[11]
Oak (*Quercus* sp.) and Chestnut (*Castanea* sp.)	Vescalagin		[12,13]
	Castalagin		
Sumac (*Rhus semialata*)	Pentagalloyl-glucoside	Gallotannins	[13,14]
	Hexagalloyl-glucoside		
	Heptagalloyl-glucoside		
	Octagalloyl-glucoside		
	Nonagalloyl-glucoside		
	Decagalloyl-glucoside		
**Condensed Tannins**			
Tea (*Camellia sinensis*)	(-)-epicatechin (+)-catechin (-)-epigallocatechin gallate	Monomers	[15]
Cocoa (*Theobroma cacao*)			
Apple (*Malus pumila*)			
Grapes (*Vitis vinifera*)			
Berries (*Vaccinium* sp.)			
Peanut (*Arachis hypogaea*)			
Persimmon (*Diospyros lotus*)			
Plums (*Prunus* sp.)	Proanthocyanidin B-Type	Dimers	[16]
Avocado (*Persea americana*)	Proanthocyanidin A-type		
Cinnamon (*Cinnamomum* sp.)	Procyanidin C1, C2	Trimers	
	Arecatanin A2 Cinnamtannin A2	Tetramers	

**Table 3:** Chemical features of gut-biotransformed tannin metabolites vs. biological effects. The table details a short list of molecular motifs of gut-biotransformed tannin metabolites.

Biological Effect	Substructure	Metabolite
Anti-inflammatory	coc(=O)c(c)c	Ellagic acid
	cC(=O)O	Gallic acid
	cc(=O)oc(c)c	Urolithin B
	ccc(cc)C(=O)O	Vanillic acid
	cc(=O)oc(c)c	Urolithin A
Antioxident	coc(=O)c(c)c	Ellagic acid
	cc(O)c(CC)c(c)O	Gallocatechin
	ccc(cc)C(=O)O	Gallic acid
	ccc	Vanillic acid
Anticancer	cc(O)cc(c)c	Ellagic acid
	cc(C)cc(c)O	5-(3′,5′-dihydroxyphenyl)-gamma-valerolactone
	cc(C)cc(c)O	5-(3′,4′,5′-trihydroxyphenyl)-gamma-valerolactone
	ccc(cc)C(=O)O	Gallic acid
	cc(C)cc(c)O	4-Hydroxy-5-(3,4,5-trihydroxyphenyl)valeric ac...
	cc(=O)oc(c)c	Urolithin C
	cccc(c)C	Vanillic acid
	cc(=O)oc(c)c	Urolithin A
Anti-atherosclerotic	cc(O)cc(c)c	Urolithin A
	CCCC(=O)O	5-(3′,4′-Dihydroxyphenyl)-gamma-valerolactone

## Supplementary Materials

The following are available online at https://www.mdpi.com/article/10.3390/microorganisms9050965/s1, Supplementary file: Table S1: Predictive Model Performances (means of ROC-AUC-score);Table S2: Neural network architecture and parameters; Figure S1: Chemical features of gut-biotransformed tannin metabolites vs. anti-inflammatory; Figure S2: Chemical features of gut-biotransformed tannin metabolites vs. antioxidant health effect; Figure S3: Chemical features of gut-biotransformed tannin metabolites vs. anticancer health effect; and Figure S4: Chemical features of gut-biotransformed tannin metabolites vs. anti-atherosclerotic health effect.
