# Supplementary material for: Effect of Gut Microbiota Biotransformation on Dietary Tannins and Human Health Implications
Source: Microorganisms. 2021 Apr 29;9(5):965. doi: 10.3390/microorganisms9050965 (PMC8145700; doi:10.3390/microorganisms9050965)
Supplement: Supplementary file 1 [file microorganisms-09-00965-s001.zip › microorganisms-1170336-supplementary.pdf]

Effect of Gut Microbiota Biotransformation on Dietary Tannins and Human Health Implications

Ibrahim E. Sallam [1], Amr Abdelwareth [2], Heba Attia [3], Ramy K. Aziz [3,4], Masun Nabhan Homsi [5], Martin von Bergen [5,6,*] and Mohamed A. Farag [7,*]

[1] Pharmacognosy Department, Faculty of Pharmacy, October University for Modern Sciences and Arts (MSA), 12566 6th of October City, Egypt; iezz@msa.eun.eg
[2] Chemistry Department, School of Sciences & Engineering, The American University in Cairo, 11835 New Cairo, Egypt; amr.abdelwareth@aucegypt.edu
[3] Department of Microbiology and Immunology, Faculty of Pharmacy, Cairo University, 11562 Cairo, Egypt; heba.mohamed@pharma.cu.edu.eg (H.A.); ramy.aziz@gmail.com (R.K.A.)
[4] Microbiology and Immunology Research Program, Children's Cancer Hospital Egypt 57357, 11617 Cairo, Egypt
[5] Helmholtz-Centre for Environmental Research-UFZ GmbH, Department of Molecular Systems Biology, 04318 Leipzig , Germany; masun.homsi@ufz.de
[6] Institute of Biochemistry, Faculty of Life Sciences, University of Leipzig, Talstraße 33, 04103 Leipzig, Germany
[7] Pharmacognosy Department, Faculty of Pharmacy, Cairo University, 11562 Cairo, Egypt
* Correspondence: martin.vonbergen@ufz.de (M.v.B.); mohamed.farag@pharma.cu.edu.eg (M.A.F.)

Supplementary File

Contents:

Table S1: Predictive Model Performances (means of ROC-AUC-score)

Table S2: Neural network architecture and parameters

Figure S1: Chemical features of gut-biotransformed tannin metabolites vs. anti-inflammatory health effect

Figure S2: Chemical features of gut-biotransformed tannin metabolites vs. antioxidant health effect

Figure S3: Chemical features of gut-biotransformed tannin metabolites vs. anticancer health effect

Figure S4: Chemical features of gut-biotransformed tannin metabolites vs. anti-atherosclerotic health effect

Table S1. Predictive Model Performances (means of ROC-AUC-score)

Biological Effect	Training (%)	Testing (%)
Anti-Inflammatory	99.81	83.28
Antioxidant	99.93	83.42
Anticancer	99.90	88.83
Anti-atherosclerotic	99.99	86.44

Table S2. Neural network architecture and parameters

Classifier	dc.models.MultitaskClassifier
n_features	1024
layer_sizes	[500, 1000]
dropouts	[.1]
learning_rate	0.001
weight_init_stddevs	[0.2]
batch_size	311
optimizer	Adam(beta1=0.9, beta2=0.999)

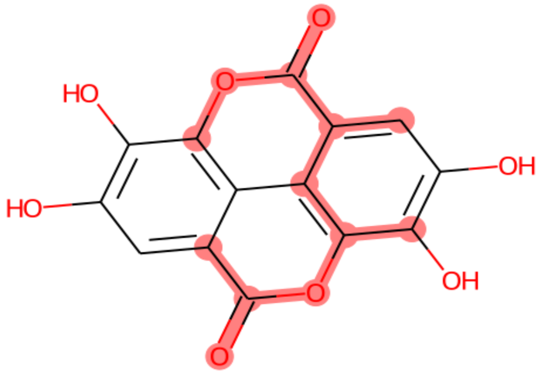

Ellagic acid

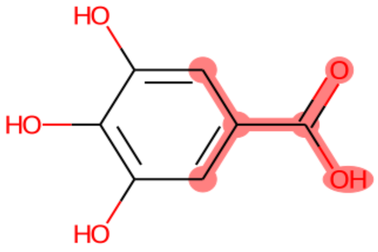

Gallic acid

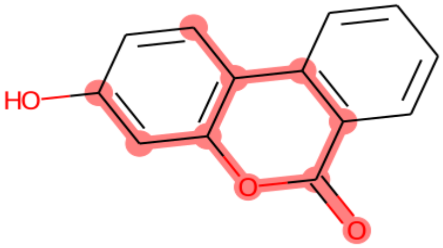

Urolithin B

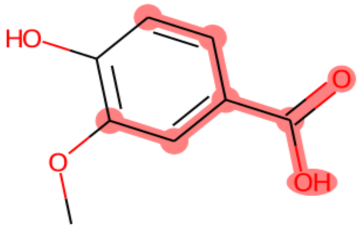

Vanillic acid

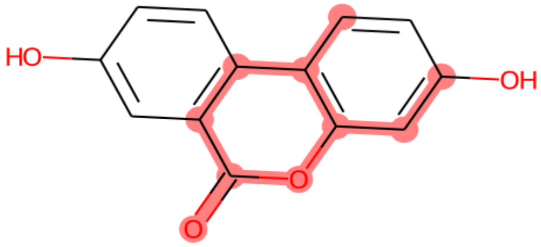

Urolithin A

Figure S1. Chemical features of gut-biotransformed tannin metabolites vs. anti-inflammatory health effect

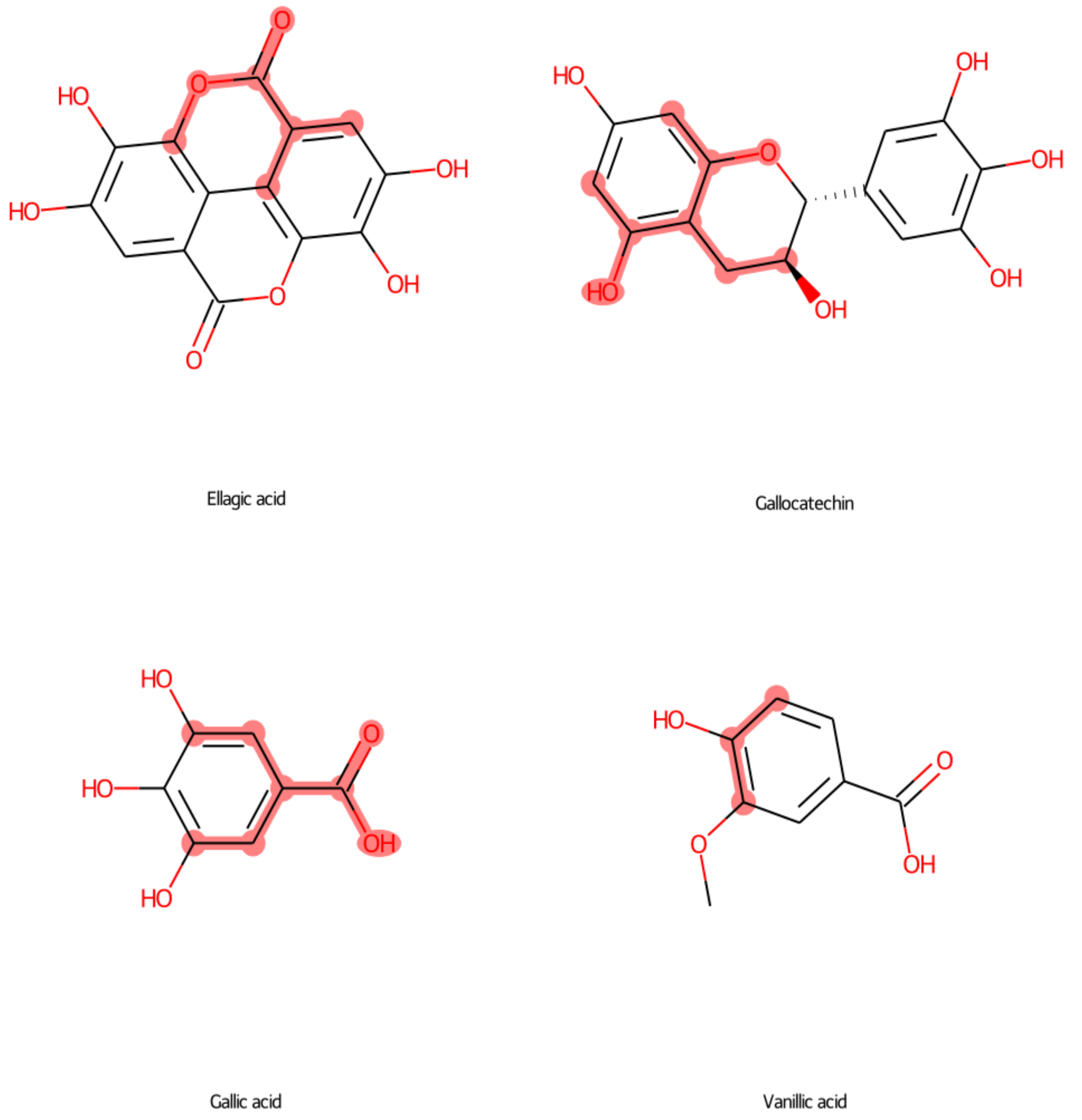

Figure S2. Chemical features of gut-biotransformed tannin metabolites vs. antioxidant health effect

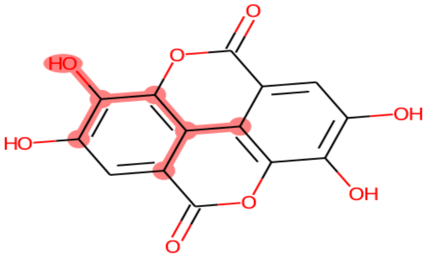

Ellagic acid

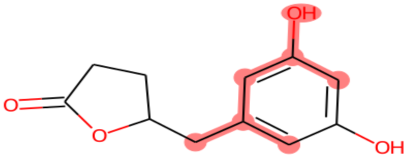

5-(3',5'-dihydroxyphenyl)-gamma-valerolactone

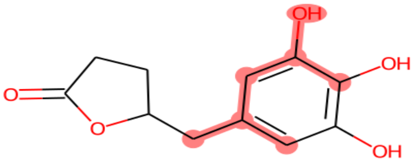

5-(3',4',5'-trihydroxyphenyl)-gamma-valerolactone

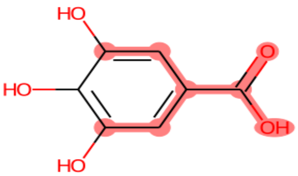

Gallic acid

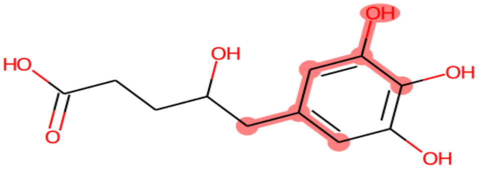

4-Hydroxy-5-(3,4,5-trihydroxyphenyl)valeric acid

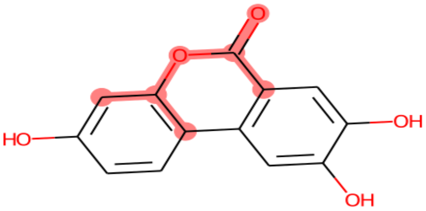

Urolithin C

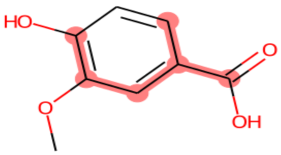

Vanillic acid

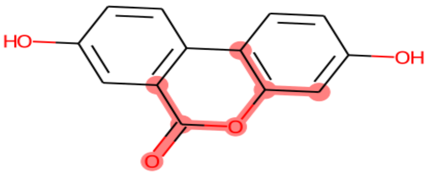

Urolithin A

Figure S3. Chemical features of gut-biotransformed tannin metabolites vs. anticancer health effect

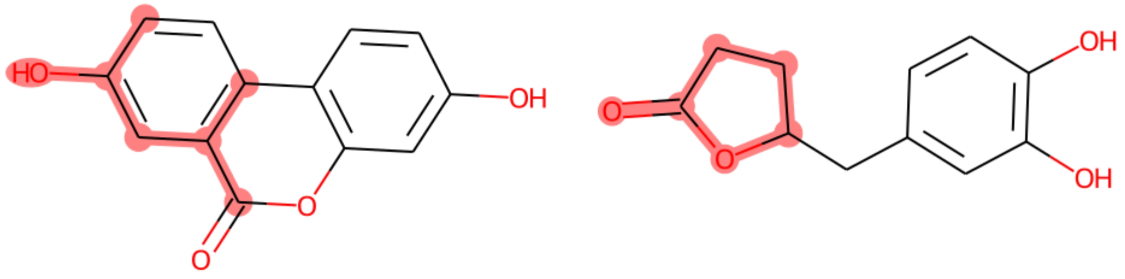

Urolithin A

5-(3',4'-Dihydroxyphenyl)-gamma-valerolactone

Figure S4. Chemical features of gut-biotransformed tannin metabolites vs. anti-atherosclerotic health effect
